# The value of pregnancy-related factors in the prediction of cardiovascular disease: a systematic review

**DOI:** 10.1016/j.ijcrp.2025.200483

**Published:** 2025-08-05

**Authors:** Zhixun Yang, Hendrikus J.A. van Os, Janet M. Kist, Rimke C. Vos, Hedwig M.M. Vos, Niels H. Chavannes, Annelieke H.J. Petrus

**Affiliations:** a Department of Public Health & Primary Care, Leiden University Medical Center, Albinusdreef 2, Leiden, 2333, ZA, the Netherlands; b Health Campus The Hague, Leiden University Medical Center, Albinusdreef 2, Leiden, 2333, ZA, the Netherlands; c National eHealth Living Lab, Leiden University Medical Center, Albinusdreef 2, Leiden, 2333, ZA, the Netherlands

**Keywords:** Pregnancy, Cardiovascular disease, Women, Female, Prediction model

## Abstract

**Aims:**

Pregnancy-related factors are associated with an increased risk of cardiovascular disease (CVD) and may help identify women at high cardiovascular risk. This study aims to provide an overview of prediction models for CVD which included pregnancy-related factors and to evaluate the impact of these factors on model performance.

**Methods:**

PubMed and Embase were systematically searched until March 2023 for studies reporting on the development or validation of prediction models for CVD which included pregnancy-related factors. Data extraction was performed using the CHARMS checklist. Risk of bias was assessed using PROBAST.

**Results:**

Seven studies were included. C-indices ranged between 0.63 and 0.79. Adding pregnancy-related factors resulted in improved C-index in four studies, ranging from 0.0033 (95 % confidence interval [CI]: 0.0022–0.0051) to 0.004 (95 % CI: 0.002–0.006). Net reclassification improvement (NRI) for events was improved in two studies, ranging from 0.01 (95 % CI: 0.003–0.02) to 0.038 (95 % CI: 0.003–0.074). NRI for non-events was improved in three studies, ranging from 0.002 (95 % CI: 0.0001–0.005) to 0.02 (95 % CI: 0.001–0.04). Two studies showed both low risk of bias and low concern regarding applicability. Subgroup analyses by age in three studies indicated larger improvements in model performance in younger women.

**Conclusion:**

Addition of pregnancy-related factors results in limited improvements in performance of CVD prediction models, with relatively larger improvements in younger women.

## 1 Introduction

Cardiovascular disease (CVD) is the main cause of death in women in Europe, accounting for 47 % of all mortalities [1]. To reduce this burden of CVD, it is crucial to identify women at high risk of CVD in an early stage, and to start preventive measures and early treatment of cardiovascular risk factors.

Prediction models are commonly used in clinical practice to identify individuals at increased risk of CVD [2,3]. However, most models are based only on traditional cardiovascular risk factors such as hypertension, hypercholesterolemia, and diabetes, without including sex-specific factors [4]. Previous studies have shown that these models tend to underestimate the CVD risk in women, especially those at younger age, which may lead to undertreatment [[5], [6], [7]].

Pregnancy is considered a cardiovascular stress test which may help to identify women with an increased risk for CVD [8]. Indeed, women who develop pregnancy complications such as hypertensive disorders of pregnancy (HDP), preterm delivery, and gestational diabetes, are found to be at an increased risk of CVD later in life [9,10]. Therefore, prediction models that include pregnancy-related factors may be able to estimate the risk for CVD in women more accurately and could be helpful to better guide the follow-up and management of high-risk women.

The aim of this systematic review is to provide an overview of models that include pregnancy-related factors for the prediction of incident CVD in women, and to assess the impact of pregnancy-related factors on the predictive performance of these models.

## 2 Methods

This systematic review was conducted according to the Preferred Reporting Items for Systematic Reviews and Meta-Analyses (PRISMA; checklist provided in Table S1).

### 2.1 Literature search

PubMed and Embase were searched from the date of inception until March 30, 2023. The detailed search strategy is shown in Appendix S1.

Studies were eligible for inclusion if they reported on the development or validation of at least one multivariable model predicting incident CVD in women. Additional eligibility criteria were a study population consisting of a population-based sample of either only parous women or a combination of nulliparous and parous women, inclusion of at least one pregnancy-related factor as predictor, and using CVD as the primary outcome (defined as any cardiovascular endpoint including at least one major cardiovascular event, i.e., acute myocardial infarction, stroke, or cardiovascular death). Finally, only studies available as full-text publications in English were eligible for inclusion.

Two reviewers (ZY and AP) independently screened the titles and abstracts of all articles and selected potentially eligible studies. Full-text records of eligible studies were assessed to decide on final inclusion. Any discrepancies between the two reviewers were solved through discussion, or by consulting a third reviewer (HvO).

### 2.2 Data extraction

Data from the included studies were extracted by two reviewers (ZY and AP) independently using a checklist for critical appraisal and data extraction for systematic reviews of prediction modelling studies (CHARMS) [11]. Information extracted included the name and country of the first author, data source, population size, definition of the CVD outcome, number of outcome events, follow-up duration, factors used in model development (separately for the model with and without pregnancy-related factors), and model performance (calibration, discrimination, and reclassification).

### 2.3 Data synthesis and analysis

We opted to conduct a narrative synthesis of the extracted evidence due to the heterogeneity in the risk factors, outcomes, and characteristics of the study populations in all the included studies. To assess the impact of pregnancy-related factors for the prediction of CVD, the predictive performance of the reference model (i.e. the model without pregnancy-related factors) was compared to that of the final model (i.e. the model including pregnancy-related factors). Measures for model performance included calibration plot, Greenwood–Nam–D'Agostino (GND) test, C-index, net reclassification improvement (NRI), and integrated discrimination improvement (IDI). The improvement of C-index in the final model compared to the reference model was defined as C-index difference. We presented the results of these measures as originally demonstrated in the articles without rounding the numbers. For all measures the 95 % confidence interval (CI) was presented; the p-value was shown only when the 95 % CI was not provided in the original article.

The risk of bias and applicability of each included study was assessed by two reviewers (ZY and HvO) according to the prediction model risk of bias assessment tool (PROBAST) [12,13]. Based on the definition of PROBAST, risk of bias occurs when shortcomings in study design, conduct, or analysis could lead to systematically distorted estimates of a model's predictive performance. Concerns regarding model applicability arise when the derivation cohort, predictors, or outcomes of the included article differ from those specified in the aim of the review. PROBAST consists of four domains (i.e., participants, predictors, outcome, and analysis) and 20 signalling questions to facilitate the assessment. Each signalling question can be answered as “yes”, “probably yes”, “no”, “probably no”, or “no information”. Based on the answers to the signalling questions, the risk of bias and concerns regarding applicability were rated as low, high, or unclear.

## 3 Results

**Fig. 1:**
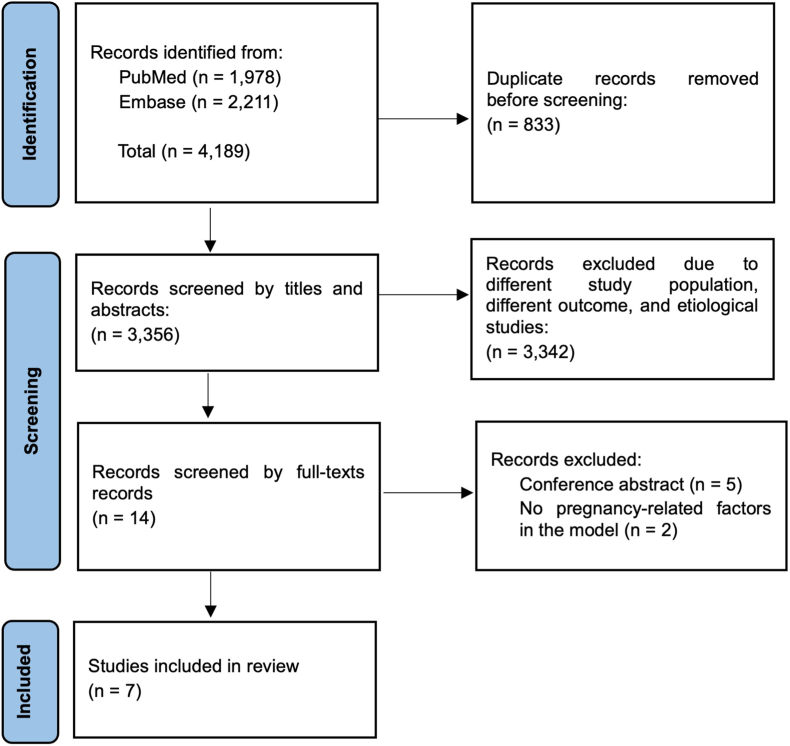
PRISMA flowchart for the literature screening.

The titles and abstracts of 3356 references were screened, of which 14 were selected for full-text screening. Seven references were excluded during full-text screening (five were only available as conference abstract [[14], [15], [16], [17], [18]], two did not include pregnancy-related factors in the model [19,20]). Finally, seven articles describing the development of at least one prediction model for incident CVD which included pregnancy-related factors were included. The study selection is displayed in Fig. 1. We did not find any article on the external validation of a prediction model with pregnancy-related factors as predictor. All included articles were published between the years 2016 and 2022.

### 3.1 Study setting and population

The studies by Stuart et al. [21] and Tanz et al. [22] were both based on data from the Nurses' Health Study II. Stuart et al. included 67,406 women and developed three separate models for the overall study population, women aged 40–49, and women aged 50–59 years. Tanz et al. included 76,512 women and developed three models as well, focusing on 10-year risk prediction for women 40 years of age, 20-year risk prediction for women 40 years of age, and 20-year risk prediction for women 30 years of age. Parikh et al. used data of 72,982 women from the Women's Health Initiative Observational Study, a cohort of both nulliparous and parous women aged 50–79 years [23]. Ukah et al. used data from the Maintenance and Use of Data for the Study of Hospital Clientele registry and included 95,537 women aged 18–45 years after a first pregnancy complicated by HDP [24]. The study by Saei Ghare Naz et al. was based on data of 4013 parous women aged 30–70 years from the Tehran Lipid and Glucose Study, a community-based cohort study, to develop a prediction model for the 15-year risk of CVD [25]. The study by Markovitz et al. was based on data of 18,231 parous women aged ≥40 years from the Nord-Trøndelag Health Study (the HUNT Study), linked to the Medical Birth Registry of Norway, local hospital records, and the Norwegian Cause of Death Registry [26]. Timpka et al. used primary care data of 11,110 parous women from the Västerbotten Intervention Program linked to data from the Swedish Medical Birth Register, Swedish National In-Patient Register, and Swedish Cause of Death Register [27]. Two separate models were developed for women aged 50 and women aged 60. Detailed information of each study is presented in Table 1.

**Table 1:** Characteristics, outcome definition, and follow-up duration in the included studies.

Authors, Country	Data source	Population size (n)	Primary outcome	Number of outcome events	Follow up duration
Stuart et al., U.S.	Cohort study	67,406	Nonfatal MI, fatal CHD, nonfatal or fatal stroke	685 (1.0 %)	n/p
Tanz et al., U.S.	Cohort study	76,512	Nonfatal MI, fatal CHD, nonfatal or fatal stroke	816 (1.1 %)	n/p
Parikh et al., U.S.	Cohort study	72,982	Fatal and nonfatal CHD	4607 (6.3 %)	Median (IQR): 12.0 (8.3–13.7) years
Ukah et al., Canada	Cohort study	95,537	CVD events or all-cause in-hospital mortality < age 60	1585 (1.6 %)	1,401,084 person years
Saei Ghare Naz et al., Iran	Cohort study	4,013	Fatal and nonfatal stroke, MI, unstable angina, CVD death, CHD, fatal coronary artery diseases, TIA, or cerebrovascular death	261 (6.5 %)	Median (IQR): 15 (12–16) years
Markovitz et al., Norway	Cohort study linked to registry data	18,231	Nonfatal MI, fatal CHD, non-fatal or fatal stroke	965 (5.3 %)	Median (IQR): 8.2 (7.4–11.1) years
Timpka et al., Sweden	Cohort study linked to registry data	11,110	MI, angina, stroke, and TIA	662 (6.0 %)	n/p

MI, myocardial infarction; TIA, transient ischemic attack; CHD, coronary heart disease; IQR, interquartile range; n/p, not provided.

### 3.2 Primary outcome and pregnancy-related factors

The descriptions of the specific definitions of the primary outcome measure, number of events, and follow-up duration of each study are shown in Table 1. The pregnancy-related factors included in the models differed per study and are specified in Table 2. HDP was the most frequently included pregnancy-related factor.

**Table 2:** Reference model, pregnancy-related factors, and measures for discrimination in the included studies.

	Reference model	Pregnancy-related factors in final model	C-index (95 % CI)			
			Population	Reference model	Final model	Difference
Stuart et al.	Pooled cohort equation	HDP and parity	Overall Age 40-49 Age 50-59	0.691 0.669 0.677	0.693 0.673 0.677	0.002 (p = 0.31) 0.005 (p = 0.29) 0.000 (p = 0.90)
Tanz et al.	Pooled cohort equation	Preterm delivery and parity	10-year prediction age ≥40 20-year prediction age ≥40 20-year prediction age ≥30	0.69 0.66 0.69	0.69 0.67 0.69	0.002 (−0.001 – 0.004) 0.004 (−0.005 – 0.009) 0.004 (0.001–0.008)
Parikh et al.	Developed by authors	Maternal age at first birth, number of still births, number of miscarriages, and breastfeeding ≥1 month	Overall	0.726	0.730	0.0033 (0.0022–0.0051)
Ukah et al.	n/p	Gestational age at delivery, caesarean section, previous complications, HDP, and number of days admitted to the neonatal intensive care unit	Overall	n/p	0.66 (0.65–0.67)	n/p
Saei Ghare Naz et al.	Framingham score	Abortion, stillbirth, pregnancy-induced hypertension/preeclampsia, and gestational diabetes	Overall	0.7798 (0.7602–0.7974)	0.7851 (0.7677–0.8041)	P < 0.001
Markovitz et al.	NORRISK 2	Pre-eclampsia, gestational hypertension, preterm delivery, and small for gestational age	Overall	0.789	0.793	0.004 (0.002–0.006)
Timpka et al.	Previously developed model	Low birth weight	Age 50 Age 60	0.69 (0.66–0.72) 0.63 (0.61–0.66)	0.70 (0.66–0.73) 0.63 (0.61–0.66)	0.01 (−0.0003 – 0.02) 0.0004 (−0.0005 – 0.0012)

HDL-C, high-density lipoprotein cholesterol; HDP, hypertensive disorders of pregnancy; DM, diabetes mellitus; n/p, not provided. Reference model: the reference model included traditional cardiovascular factors. Final model: the final model consisted of the described pregnancy-related factors added to the reference model. Difference: the improvement of C-index in the final model compared with the reference model.

### 3.3 Model development and performance

Markovitz et al. used the Fine and Gray competing risk analysis to develop the model, accounting for non-cardiovascular death as competing event. The other studies used the Cox proportional hazards model. Internal validation was performed in all studies using bootstrapping. Saei Ghare Naz et al. predicted the 15-year CVD risk, Tanz et al. predicted both 10-year and 20-year risk, while all other studies predicted the 10-year risk. A calibration plot was provided by Stuart et al., Ukah et al., Saei Ghare Naz et al., and Markovitz et al., all indicating good calibrations. The GND test by Tanz et al. showed sufficient calibration in the two models for 20-year risk prediction, but not in the model for 10-year prediction. Timpka et al. also performed a GND test and reported adequate calibration. Calibration information was not provided by Parikh et al.

The C indices of all final models in the included articles ranged from 0.63 to 0.79 (Table 2). Ukat et al. developed a model without comparing it to any reference model. In the other six studies, the C indices of four final models were significantly improved after the addition of pregnancy-related factors, which were developed by Tanz et al. (20-year prediction, age ≥30), Parikh et al., Saei Ghare Naz et al., and Markovitz et al.

**Fig. 2:**
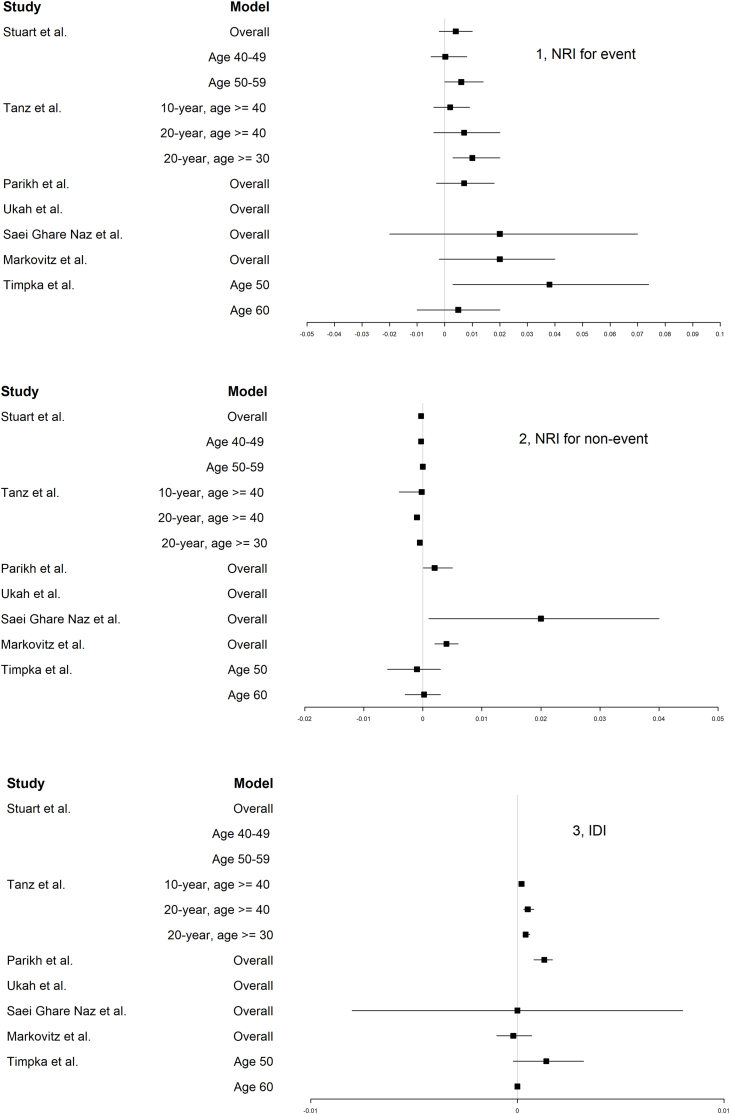
NRI and IDI of the models in the included studies. NRI, net reclassification index; IDI, integrated discrimination index. In the study by Stuart et al., IDI was not presented because 95 % CI was not provided. The values of IDI in the model for overall population, women aged 40–49 years, and women aged 50–59 years was 0.0002 (p < 0.001), 0.0004 (p < 0.001), and 0.00006 (p = 0.04), respectively. In the study by Ukah et al., In the study by Ukah et al., only one model was developed without any comparison with an existing model.[Not Available in CrossRef][Not Available in Internal Pubmed][Not Available in External Pubmed].

Fig. 2 presents the values of NRI and IDI which are measures for reclassification. NRI for events was significantly improved in the studies by Tanz et al. (0.01 [95 % CI: 0.003–0.02] for the model with 20-year prediction, age ≥30) and Timpka et al. (0.038 [95 % CI: 0.003–0.074] for the model at age 50). NRI for non-events was significantly improved in the studies by Parikh et al., Saei Ghare Naz et al., and Markovitz et al. The value of NRI for non-events was 0.002 (95 % CI: 0.0001–0.005), 0.02 (95 % CI: 0.001–0.04), and 0.004 (95 % CI: 0.002–0.006), respectively. IDI was significantly improved in three studies. In the study by Stuart et al., IDI was 0.0004 (p < 0.001) in women aged 40–49 years and 0.00006 (p = 0.04) in women aged 50–59 years. In the study by Tanz et al., IDI was significant in all three models, ranging from 0.0002 (0.0001–0.0003) to 0.0005 (0.0003–0.0008). In the study by Parikh et al., IDI was 0.0013 (0.0008–0.0017).

### 3.4 Subgroup analysis by age

Three of the included articles performed subgroup analysis and evaluated the added value of pregnancy-related factors in different ages. The models for younger women performed better in at least one measure for discrimination or reclassification in all three studies. In the study by Stuart et al., IDI in women 40–49 years old was larger than that in women 50–59 years old. In the study by Tanz et al., C index difference and NRI for events were only significant in the 20-year risk prediction model for women age ≥30, but not for the two models for women aged ≥40. In the study by Timpka et al., NRI for events was significant in the model for women aged 50 but not in the model for women aged 60.

### 3.5 Risk of bias assessment

The risk of bias and applicability of each included study was assessed according to PROBAST. Only the studies by Ukah et al. and Markovitz et al. showed low risk of bias and low concern regarding applicability. The study by Stuart et al. and Tanz et al. showed high risk of bias and unclear concern regarding applicability. The other three studies showed high risk of bias and low concern regarding applicability. Detailed information on PROBAST and the assessment of the risk of bias and applicability for each study can be found in Table S2.

## 4 Discussion

### 4.1 Main findings

In this systematic review, we included seven articles reporting on prediction models for incident CVD that included pregnancy-related factors. The C-indices of all models indicated moderate to good discrimination. One article developed a prediction model which included pregnancy-related factors but did not compare it to a reference model. The other six studies compared the final model to a reference model, and all showed statistically significant but small improvements in one or more measures for discrimination and reclassification. In the three studies with subgroup analyses for different ages, adding pregnancy-related factors resulted in larger improvements of model performance in younger populations. Only two articles showed both low risk of bias and low concern regarding applicability.

### 4.2 Interpretation of results

#### 4.2.1 The added value of pregnancy-related factors in the prediction of CVD

In this review, we assessed the impact of pregnancy-related factors on the performance of prediction models for incident CVD in women and found no or only minor improvements in model performance. The minor improvements in C-indices may be explained by the fact that discrimination of the reference models was already moderate to good, limiting the potential improvement regardless of the predictive ability of new predictors [28]. In addition, the C-index is relatively insensitive to change [29]. Therefore, reclassification measures such as NRI and IDI are more suitable for evaluating the impact of novel predictors. However, these measures also showed only small improvements, which may be explained by several reasons. First, due to the low absolute risk of CVD in all articles (1.0–6.5 %), only few participants could be reclassified from a lower into a higher risk category after adding pregnancy-related factors [30], limiting the potential improvement of reclassification measures. Second, the prevalence of predictors can influence the improvement in NRI and IDI. Therefore, limited added predictive value may be observed for predictors with a strong statistical association but low prevalence [31,32]. Finally, collinearity between newly added pregnancy-related factors and traditional cardiovascular risk factors, e.g. hypertension, diabetes, and hypercholesterolemia, may explain the limited improvement in reclassification measures [28].

#### 4.2.2 Prediction models in different age groups

The three studies with subgroup analyses for different age groups showed larger improvements in discrimination and reclassification in younger populations [21,22,27]. This may be explained by the mediating effect of traditional cardiovascular risk factors. Previous studies showed that the development of chronic hypertension, diabetes, and hypercholesterolemia in women with a history of pre-eclampsia and gestational hypertension accounted for 57 % and 84 % of the increased CVD risk, respectively [33]. In line with these findings, development of diabetes mellitus in women with a history of gestational diabetes accounted for 20 % of the increased CVD risk [34]. These mediating effects of traditional cardiovascular risk factors may attenuate the predictive value of pregnancy-related factors. Since the prevalence of traditional cardiovascular risk factors increases with age, this effect will be more pronounced in older women. This may explain the observed different model performance among different age groups.

### 4.3 Clinical implications

Prediction models for CVD are frequently used to identify high-risk individuals to target cardiovascular risk management more precisely. Since pregnancy-related factors are associated with the development of CVD in women, we hypothesized that adding these factors to a prediction model might improve the identification of high-risk women [9,10]. The improvements in NRI observed in five included studies indicate that an additional 1.0–3.8 % of women who developed CVD were reassigned to a higher risk category while an additional 0.2–2.0 % of women who did not develop CVD were reassigned to a lower risk category. Although these reclassifications are small, they may still impact cardiovascular risk management on a population level when medical resources of the 0.2–2.0 % low-risk women would be reallocated, and an additional 1.0–3.8 % of young high-risk women would receive more adequate follow-up and treatment to prevent CVD. Furthermore, these small improvements may be enough to justify the inclusion of pregnancy-related factors in CVD prediction models, as these factors can be collected easily based solely on medical history, and at low cost.

### 4.4 Future directions

Pregnancy-related factors generally manifest when women are in their 20s or 30s. As such, pregnancy enables early identification of women at increased CVD risk and provides a window of opportunity to optimize cardiometabolic health after pregnancy to prevent or delay development of CVD. Furthermore, pregnancy can be considered a teachable moment, when women may be more receptive to improve their lifestyle and health behaviours and generally have an increased frequency of contact with healthcare professionals [35]. However, in this systematic review, we identified only one article predicting the risk of CVD immediately after pregnancy and this study focused only on women with a pregnancy complicated by HDP rather than taking into account all pregnancy-related factors [24]. Current cardiovascular risk models generally focus on a prediction horizon of 10 years [36]. However, absolute 10-year CVD risk is low in young women, even if they have unfavourable CVD risk profiles, which may lead to underestimation of their CVD risk [[37], [38], [39]]. Lifetime prediction may provide more valuable information to correctly estimate the risk of CVD especially in young women [40], while prediction models using hypertension as an intermediate endpoint may be another suitable alternative to identify high-risk women early in the life course [41]. Finally, we did not find any articles on the external validation or implementation of CVD prediction models including pregnancy-related factors. These studies are needed to determine the true clinical benefit of these models [42].

### 4.5 Strengths and limitations

This review provides an up-to-date overview of prediction models for CVD that include pregnancy-related factors and evaluates the added value of pregnancy-related factors in these models. A risk of bias assessment was performed using the latest guidelines [12,13]. Several limitations need to be considered. First, pooled quantitative analyses were not performed due to the low number of included studies and their heterogeneity in terms of study populations, predictors, and outcome definitions. Second, six out of seven included studies were carried out in developed countries, limiting the generalizability of results to other populations. Finally, since the coefficients of the models in the included articles were seldom provided, we could not distinguish which pregnancy-related factor may improve the predictive performance the most.

## 5 Conclusion

In this review, we included seven articles on the development of prediction models for incident CVD which included pregnancy-related factors. The addition of pregnancy-related factors to the reference models led to statistically significant but small improvements in model performances, with relatively larger improvements observed in younger women. The clinical utility of pregnancy-related factors in current CVD prediction remains limited. Future research should investigate whether these factors might prove more valuable in lifetime CVD risk prediction models, or models targeting intermediate cardiovascular outcomes that may manifest earlier in the disease trajectory.

## CRediT authorship contribution statement

**Zhixun Yang:** Writing – original draft, Project administration, Investigation, Conceptualization, Writing – review & editing, Visualization, Methodology, Formal analysis. **Hendrikus J.A. van Os:** Writing – review & editing, Conceptualization, Methodology. **Janet M. Kist:** Writing – review & editing, Methodology. **Rimke C. Vos:** Writing – review & editing. **Hedwig M.M. Vos:** Writing – review & editing. **Niels H. Chavannes:** Writing – review & editing, Conceptualization. **Annelieke H.J. Petrus:** Writing – review & editing, Project administration, Investigation, Supervision, Methodology, Conceptualization.

## Funding

This systematic review has been conducted without external funding or sponsorship.

## Declaration of competing interest

The authors declare that they have no known competing financial interests or personal relationships that could have appeared to influence the work reported in this paper.
