# Supplementary material for: The value of pregnancy-related factors in the prediction of cardiovascular disease: a systematic review
Source: Int J Cardiol Cardiovasc Risk Prev. 2025 Aug 5;27:200483. doi: 10.1016/j.ijcrp.2025.200483 (PMC12358648; doi:10.1016/j.ijcrp.2025.200483)
Supplement: Multimedia component 2 [file mmc2.docx]

Searching strategy in PubMed.

| Search | Query |
| --- | --- |
| 1 | "cardiovascular diseases/adverse effects"[Majr] OR "Cardiovascular Diseases/complications"[Majr] OR cardiovascular disease*[tiab] OR cardiovascular[tiab] OR "Ischemic Attack, Transient"[Majr] OR ischemic attack[tiab] OR "Stroke"[Majr] OR "Stroke*"[tiab] OR Cerebrovascular accident*[tiab] OR "Myocardial Infarction"[Majr] OR myocardial infarction*[tiab] OR heart disease*[tiab] |
| 2 | "Risk Assessment"[majr] OR "Risk Assessment"[tiab] OR risk score*[tiab] OR Risk Analysis*[tiab] OR predict*[tiab] OR risk prediction*[tiab] OR risk tool*[tiab] OR model predicting[tiab] OR risk stratification [tiab] |
| 3 | ((("Pregnancy"[Majr] OR "Pregnant Women"[Majr] OR "Pregnan*"[tiab] OR gravidit*[tiab] OR gestation*[tiab]) AND (Complication*[tiab] OR "adverse effects" [Subheading] OR "adverse effect*"[tiab])) OR "Pregnancy Complications"[Majr] OR "Pregnancy Complications, Cardiovascular"[Majr] OR "Pregnancy Complication*"[tiab] OR "Pregnan*"[ti] OR gestational diabetes[tiab] OR "Diabetes, Gestational"[Majr] OR preterm deliver*[tiab] OR "Premature Birth"[Majr] OR (hypertensive[tiab] AND disorder*[tiab] AND pregnan*[tiab])) OR "small for gestational age"[tiab] OR "Infant, Low Birth Weight"[Majr] OR "Low Birth Weight"[tiab] OR "reproductive history"[tiab] OR "Reproductive History"[Majr] |
| 4 | #1 AND #2 AND #3 |

Searching strategy in Embase.

| Search | Query |
| --- | --- |
| 1 | *pregnancy/ or *pregnant woman/ or ((Pregnan* or gravidit* or gestation*) adj3 (Complication* or adverse effect*)).ti,ab,kw. or *adverse event/ or *pregnancy complication/ or *pregnancy diabetes mellitus/ or *prematurity/ or (gestational diabetes or preterm deliver*).ti,ab,kw. or (hypertens* and disorder* and pregnan*).ti,ab,kw. or *small for date infant/ or *low birth weight/ or *reproductive history/ or (small for gestational age or Low Birth Weight or reproductive history).ti,ab,kw. |
| 2 | risk assessment/ or ("Risk Assessment" or risk score* or Risk Analysis* or predict* or risk prediction* or risk tool* or model predicting or risk stratification).ti,ab,kw. |
| 3 | *cardiovascular disease/si |
| 4 | *cardiovascular disease/co |
| 5 | *transient ischemic attack/ or *cerebrovascular accident/ or (cardiovascular disease* or isch?emic attack* or Stroke* Cerebrovascular accident* or myocardial infarction* or heart disease*).ti,ab,kw. |
| 6 | 3 or 4 or 5 |
| 7 | 1 and 2 and 6 |
